# Chikungunya antibodies detected in non-human primates and rats in three Indian Ocean islands after the 2006 ChikV outbreak

**DOI:** 10.1186/1297-9716-45-52

**Published:** 2014-05-01

**Authors:** Gwenaël Vourc’h, Lénaïg Halos, Amélie Desvars, Franck Boué, Michel Pascal, Sylvie Lecollinet, Stéphan Zientara, Thomas Duval, Angella Nzonza, Michel Brémont

**Affiliations:** 1 Institut National de la Recherche Agronomique (INRA), UR346 Epidémiologie Animale, Saint-Genès-Champanelle, France; 2 Agence Nationale de Sécurité Sanitaire (ANSES), Laboratoire de la rage et de la faune sauvage de Nancy, Malzéville, France; 3 INRA, UMR 0985 Écologie et Santé des Écosystèmes, Rennes, France; 4 ANSES, Laboratoire de Santé Animale, UMR 1161 Virologie, Maisons-Alfort, France; 5 INRA, UR892 Virologie et Immunologie Moléculaires, Jouy-en-Josas, France; 6 Present address: MERIAL, Parasiticides and Pets, Lyon, France; 7 Present address: Department of Clinical Microbiology, Umeå University, 90185 Umeå, Sweden; 8 Present address: Société Calédonienne d’Ornithologie, Antenne Nord, Galerie Goropverbe Poindimié, Nouvelle Calédonie

## Abstract

The role of terrestrial vertebrates in the epidemiology of chikungunya disease is poorly understood. We evaluated their exposure and amplification role during the 2006 chikungunya outbreak in the Indian Ocean. Blood samples were collected from 18 mammalian and reptile species from Reunion Island, Mauritius and Mayotte. Among the 1051 samples serologically tested for chikungunya virus (CHIKV), two crab-eating macaques (*Macaca fascicularis*) and two ship rats (*Rattus rattus*) proved to be exposed to CHIKV. CHIKV RNA was not detected in 791 analyzed sera. Our results confirm the preferential infection of simian primates and suggest that other vertebrates played a poor or no role in CHIKV transmission during the 2006 outbreak.

## Introduction, methods and results

Since its first description in Tanzania in 1953, chikungunya virus (CHIKV) has caused numerous outbreaks in Africa and Southeast Asia. Since 2004, the incidence and geographical distribution of CHIKV have increased dramatically, with major epidemics reported on the Indian Ocean islands, in Asia, as well as in Africa. Furthermore, autochthonous outbreaks were reported in Italy (2007) and in France (2010) [1]. CHIKV is an arbovirus belonging to the *Togaviridae* family in the genus *Alphavirus*. As an arbovirus, CHIKV is of animal origin and originates from forest non-human primates (NHP) [2]. In Africa, the virus is maintained in a sylvatic cycle involving forest dwelling *Aedes* spp. mosquitoes and wild NHP, while in Asia CHIKV seems to have a primarily human-mosquito transmission cycle which periodically causes epidemics in urban centres involving *A. aegypti* as the main vector [1]. Sylvatic transmission has not been documented in Asia, although wild NHP have been found seropositive in Malaysia and in the Philippines [3,4].

Evidence that species other than NHP could be reservoir hosts for CHIKV and their potential roles in the virus epidemiology remains unclear. In Africa, some studies reported the presence of CHIKV antibodies in rodents, birds, and reptiles, as well as in domestic mammals living in close proximity to anthropophilic mosquito habitats [5,6]. However, the serological screenings were mainly based on haemagglutination-inhibition or complement fixation tests and were rarely confirmed by the virus neutralization test which is the reference method for CHIKV antibody detection. Few virus isolations have been reported from other species than NHP, including striped ground squirrels (*Xerus erythropus*), yellow bats (*Scotophilus* sp.), golden sparrows (*Auripasser luteus*) and *Mus musculus* mice [7].

Reunion Island (Indian Ocean) underwent an unforeseen outbreak of chikungunya disease, characterized by high fever and arthralgia, during the 2005–2006 austral summer. Approximately 266 000 people out of a total of 785 000 inhabitants [8] suffered from infection between April 2005 and July 2006. The virus that reached Reunion Island in 2005 belonged to the East-Central-South-African and Indian Ocean group [9] and was a new variant efficiently transmitted by *A. albopictus*[10]. With the exception of humans and a couple of specimens in zoological gardens, Reunion Island does not host NHP whereas Mayotte and Mauritius count an important population of *Eulemur fulvus (*brown lemurs) and *Macaca fascicularis (*crab-eating macaques) respectively. Since *A. albopictus* can feed on various vertebrates in Asia and recently invaded locations including Reunion Island [11,12], vertebrates from Reunion Island may have been exposed to CHIKV during the epidemics.

**Figure 1:**
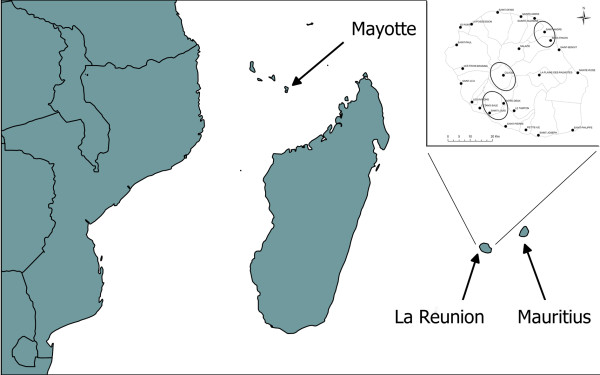
**Map of the Indian Ocean with the 3 study areas, Mayotte, Mauritius and Reunion Islands.** In total, 54 brown lemurs from Mayotte, 134 crab-eating macaques from Mauritius and 984 animals belonging to 17 species from Reunion were sampled. On Reunion (upper right corner), the 3 main sampling zones were located in the district of Saint-André (east, 322 individuals), the district of Saint-Louis (south-west, 308 individuals) and the Cirque of Cilaos (239 individuals). Ninety-four individuals originated from other areas, while 21 individuals were selected on account of an increased risk for CHIKV infection.

The aim of our study was to investigate the presence of CHIKV in terrestrial vertebrates to study the role that they may have played in the 2006 chikungunya epidemic on the Indian Ocean islands. We sampled 1172 specimens belonging to 18 wild and domestic non-primate vertebrate species from La Reunion, Mayotte and Mauritius (see Figure 1 and Table 1). Domestic species (dogs and cats) suspected to be at risk of CHIKV infection were sampled, namely symptomatic animals with arthralgia, myalgia and fever or animals belonging to patients recently diagnosed with chikungunya disease. Moreover, NHP from Reunion Island zoological garden, wild lemurs from Mayotte, and macaques from Mauritius were also sampled. Whenever possible, 40 individuals per species were trapped according to a recommended procedure. Blood samples were collected at classic puncture sites and sera were extracted after centrifugation. Kidney, spleen, and liver were sampled on euthanized rats, mice and shrews and stored at -80 °C for virus detection. This study was performed in strict accordance with the French guidelines on animal experimentation and welfare.

**Table 1:** Number of specimens tested by qRT-PCR and by ELISA, and number of seropositive animals

		**qRT-PCR**	**ELISA**	
**Species**	**Sampling location**	Number tested*	**Number tested**	**Number positive (%)**
**Domestic carnivores**				
Cat - *Felis catus*	Reunion	38	37	0
Dog - *Canis lupus*	Reunion	69	68	0
**Farm mammals and poultry** (Reunion Island)				
Horse - *Equus ferus*	Reunion	76	97	0
Cattle - *Bos primigenius*	Reunion	115	116	0
Goat - *Capra aegagrus*	Reunion	95	115	0
Sheep - *Ovis aries*	Reunion	25	49	0
Pig - *Sus scrofa*	Reunion	48	108	0
Poultry - *Gallus gallus*	Reunion	37	113	0
**Wild mammals**				
Shrew - *Suncus murinus*	Reunion	105	45	0
Ship rat - *Rattus rattus*	Reunion	74	75	3 (4.0%)
Norway rat - *Rattus norvegicus*	Reunion	6	17	0
House mouse - *Mus musculus*	Reunion	33	20	0
**Reptiles**				
Panther chameleon - *Chamaeleo pardalis*	Reunion	17	not tested	not tested
**Non-human primates**				
Brown lemur - *Eulemur fulvus*	Mayotte	53	52	2 (3.8%)
Crab-eating macaques - *Macaca fascicularis*	Mauritius	not tested	134	1 (0.7%)
Crab-eating macaques - *Macaca fascicularis*	Reunion	not tested	1	1 (100.0%)
Hamadryas Baboon - *Papio hamadryas*	Reunion	not tested	2	0
Southern Pig-tailed Macaque - *Macaca nemestrina*	Reunion	not tested	1	0
Campbell’s Monkey - *Cercopithecus campbelli*	Reunion	not tested	1	0
**Total**		**791**	**1051**	**7**

*All samples were negative by qRT-PCR.

CHIKV antibodies were identified by indirect ELISA, by virus neutralization, and by indirect immunofluorescence assays (using the CHIKV isolate 695, isolated on Reunion Island in 2005 and kindly provided by I. Leparc-Goffart, IRBA, Marseille, France). ELISA plates were coated with UV-inactivated CHIKV (10^6^ particles per well) or CHIKV cell-culture antigens provided by Institut Pasteur Lyon, France (250 ng/well); diluted serum samples (1/100) were added after completion of the blocking step and appropriate HRP-conjugated secondary antibodies (Jackson ImmunoResearch Europe Ltd, Suffolk, UK, with dilutions ranging from 1/1000 for most species to 1/100 000 for anti-cat IgG) were used. A positive control, consisting of a human positive serum (Institut Pasteur Lyon, France) was added in each ELISA plate and the ELISA cut-off was determined as the mean optical density of negative sera plus 2 SD. The presence of anti-CHIKV antibodies was monitored in 12 sera (7 ELISA positive and 5 ELISA negative) by indirect immunofluorescence assay (IFA) on Vero cells 48 h post-infection with 0.1 MOI of CHIKV. Cells were fixed and incubated with serially diluted sera before addition of fluorescein isothiocyanate-conjugates (anti-rat conjugate for ship rats, anti-monkey conjugate for lemurs and macaques, IgG(H + L), Paris, Compiègne, France) diluted 1/100 in PBS-Tween 20 0.05%. Fluorescence was monitored at magnification 40X (Zeiss microscope). Moreover, a 96-well microneutralization test was used to confirm the results obtained in ELISA for 10 sera. A serum was considered positive if Vero cells were protected at the 1/20 dilution. Sera and organs (liver, kidney and spleen) from the 3 ELISA-positive rats were also tested for the presence of CHIKV RNA. Ten μL of enterovirus nucleic acid were added to 100 μL of each serum to control the extraction process. RNA of the resulting solution was extracted using Nucleospin II RNA Macherey Nagel kit (Düren, Germany). Extracts were then analyzed by qRT-PCR with TaqMan® probes following a previously published protocol [13].

A total of 1172 animals were sampled between May 2006 and April 2007, among which 984 individuals belonging to 17 vertebrate species originating from Reunion Island, 54 brown lemurs from Mayotte and 134 crab-eating macaques from Mauritius (see Table 1). Reunion Island samples included 21 domestic animals (17 dogs, two cats and two chickens) considered at risk for CHIKV infection.

Amongst the 1051 individuals analyzed by ELISA, seven were found positive: three of the 75 ship rats (4.0%) tested from Reunion Island, two of the 52 brown lemurs from Mayotte (3.8%), one of the 134 crab-eating macaques from Mauritius (0.7%), and the one crab-eating macaque that was sampled at the Reunion Island zoo (see Table 1). The three seropositive rats were trapped in the east part near Saint-André on Reunion Island, an area where the prevalence of human infection was high. The presence of anti-CHIKV antibodies was confirmed by IFA and virus neutralization test in all the ELISA-positive crab-eating macaques and in two out of three ship rats, but could not be confirmed with the two ELISA-positive brown lemurs. Seven hundred ninety-one sera as well as organs (liver, kidney and spleen) from the seropositive rats were tested by qRT-PCR. All samples were RT-PCR negative (Table 1).

## Discussion

Our investigation of the presence of CHIKV in domestic and wild vertebrates from three Indian Ocean islands, which underwent a huge CHIKV outbreak in humans in 2005–2006, revealed that terrestrial vertebrates were unfrequently exposed to CHIKV despite the high infection rate in the human population. Exposure to CHIKV was evidenced in all NHP species, including lemurs, present on the three considered islands and only three ship rats were tested seropositive out of the 979 non-primate vertebrates sampled. CHIKV RNA was not detected in any of the analyzed sera nor in the organs of the rats testing positive. These results confirmed the preferential association of CHIKV with humans and NHP and indicate that vertebrates other than NHP and ship rats are mainly dead-end hosts for CHIKV on Reunion Island. During the 2006 epidemic in the Indian Ocean, the virus seems to have circulated among a human-mosquito cycle in a similar manner to that found in Asia [14].

Seroprevalence against CHIKV among NHP was low on Reunion, Mayotte and Mauritius. Previously crab-eating macaques had been found seropositive in Malaysia using hemagglutination-inhibiting tests [4] and in the Philippines using ELISA tests [3]. Only ten crab-eating macaques were present in the zoo of Reunion Island when this work was conducted, thus the probability that this species had an epidemiological role in virus transmission on this island is negligible. In contrast, they may have played a role on Mauritius which counts thousands of macaques [15].

To our knowledge, the present study is the first to report the presence of CHIKV antibodies in lemurs and this finding should be considered with caution. Antibodies could be evidenced by indirect ELISA only, which suggests either that ELISA is more sensitive than the IFA or virus neutralization test, as generally reported in the literature [16] or that ELISA generated false positive results with sera of lemurs. Consequently, more data should be generated to confirm potential CHIKV infections in lemurs.

Marchette et al. described for the first time CHIKV infection in rodents, with low seropositive titres in one *Rattus sabanus* out of 353 trapped in Malaysia [4]. We failed to detect the CHIKV in the organs of the three seropositive *Rattus rattus* trapped on Reunion Island. These rats may have been in contact with the virus and carried the virus, but not long enough to detect it by qRT-PCR. Mayotte, Mauritius, and Reunion Island count a high number of introduced *Rattus* spp. which are able to adapt to a great variety of habitats, climates and tropic regimens, and are accordingly widespread (pers. obs.). Consequently, it would be interesting to test experimentally whether ship rats can amplify the virus and infect *Aedes* mosquitoes through blood meals, to further characterize the role of this species in the circulation and or dissemination of CHIKV in association with mosquitoes along commercial routes in the Indian Ocean.

The absence of antibodies in most vertebrates could be due 1/ to an absence of contact with CHIKV or 2/ to an absence of immune response induction following poor CHIKV replication. A study on Reunion Island indicates that although *A. albopictus* feeds preferentially on humans, this mosquito can feed on every domestic species that we tested in our study [11]. These data support our second hypothesis. Otherwise, several studies that relied mostly on haemagglutination inhibition tests have described CHIKV antibodies in the non-primate species that we sampled: poultry [4,5], cattle [4,17], sheep [17,18], goats [4,18], pigs [4], horses [4,19,20], and dogs [4]. No prior results are available for shrews, nor mice or chameleon, and one study reported negative results in cats [21]. These divergent results could result from specificity issues with the serological tools used or from differing CHIKV circulation patterns (with transmission cycles closer to the ones documented in Asia).

We did not detect any viremia in the sampled animals. Viremia in animals has been assessed only through experimental infections. These studies reported detectable viremia in NHP [22,23], rodents [24,25], but failed to detect CHIKV in other domestic species [22]. Upon CHIKV infection, viremia lasts a maximum of seven days after inoculation [23,24]. Due to the timing of the sampling campaign, which was initiated three months after the peak in human infections, viremia may have been missed [8].

In conclusion, the present study confirms the preferential association of CHIKV with simian NHP during the 2006 outbreak in the Indian Ocean region. It suggests that other vertebrates, including NHP did not play a role in the transmission during this outbreak.

## Competing interests

The authors declare that they have no competing interests.

## Authors’ contributions

GV and MB conceived and designed the experiments. LH, AD, MP, TD took part in the sampling effort. GV, LH, AD, FB, SL, AN performed the analysis and interpreted the data. All authors contributed to the drafting and revision of the manuscript and have given their approval for publication of the latest version.
